# Foreword: Pacific Fragments

**DOI:** 10.3390/molecules21070926

**Published:** 2016-07-16

**Authors:** Daniel A. Erlanson

**Affiliations:** Carmot Therapeutics Inc., 409 Illinois Street, San Francisco, CA 94158, USA; derlanson@carmot.us; Tel.: +1-415-978-2159

*Pacific*, which is derived from the Latin *pac*, means peaceful. It is also the name of Earth’s largest ocean, which surrounds the Hawaiian island of Oahu and borders several continents. Every five years for the past three decades, delegates from countries surrounding the Pacific meet in Honolulu to discuss the latest developments in chemistry—broadly defined.

A *fragment*, which is also derived from Latin, is a small piece of something else. Fragment-based lead discovery (FBLD) involves starting with a very small molecule (typically <250 Da or so) and improving this by whatever means possible, ultimately to deliver a chemical probe or drug. This approach has several key advantages compared with more traditional means of drug discovery, such as high-throughput screening. Because there are fewer possible fragments than drug-sized molecules, one can cover a larger fraction of chemical diversity space with a smaller screening set—just a few thousand as opposed to a few million molecules. This efficiency enables discovery efforts at smaller organizations, such as start-up companies and academic laboratories. More importantly, FBLD expands the range of targets amenable for screening to proteins that have proven recalcitrant to conventional approaches. Moreover, by starting from small fragments and growing them judiciously, researchers can control properties such as molecular weight and lipophilicity, potentially leading to superior clinical candidates. Of course, these potential advantages also come with challenges, chief among them being how to find low-affinity fragments in the first place, and how to improve their affinities.

These issues filled two and a half days of discussion and posters at the 2015 International Chemical Congress of Pacific Basin Societies, or Pacifichem. The FBLD symposium was organized by Martin Scanlon (Monash University), Ke Ruan (University of Science and Technology of China), Daisuke Tanaka (Sumitomo Dainippon Pharma), Joel Tyndall (University of Otago), and me, with help and advice from Pacifichem veteran Ray Norton (Monash University). Martin and Ray were asked to compile a Special Issue of *Molecules* devoted to FBLD, and they invited the Pacifichem delegates to contribute articles. What follows is the result.

Although pacific means peaceful, the Pacific Ocean is renowned for spectacular tempests. Similarly, FBLD has taken the drug-discovery world by storm. Only twenty years ago researchers at Abbott published what is widely considered the first practical demonstration of the approach [1]. Since then fragment-based drug discovery has contributed to more than thirty drugs that have entered the clinic, two of which have already been approved by the US Food and Drug Administration (FDA). These include some very challenging targets: venetoclax, approved earlier this year, targets a protein-protein interaction, while verubecestat targets the difficult aspartyl protease beta-secretase 1 (BACE1). Both of these programs were discussed at the symposium.

FBLD has been extensively reviewed in dozens of book chapters and papers, both specific and general, including recent reviews by the Editors of this Special Issue [2,3]. In the past decade, eight books have been published on the topic, the most recent of which came out just this year [4]. I won’t attempt to review the field in this brief Foreword. Instead, I would just like to highlight two ways in which FBLD is a highly diverse endeavor, and this diversity nurtures productive collaborations.

The first aspect of diversity is scientific. Designing, assembling, and curating high-quality fragment libraries require the skills of computational, medicinal, and analytical chemists. Screening methods often involve biophysical approaches, such as X-ray crystallography, nuclear magnetic resonance (NMR), surface plasmon resonance (SPR), and more. Medicinal and computational chemists are essential for transforming low-affinity fragments to potent molecules, while biochemists and cell biologists are necessary for characterizing and validating lead compounds in increasingly complicated (and relevant) assays. Pharmacologists are indispensable once compounds are tested in animals, and disease-specific biologists are often needed to interpret results targeting proteins that have never been successfully modulated with a small molecule. In addition, all of this is before a compound is even chosen for clinical development! Drug discovery in general requires multiple skills, but this is all the more true with FBLD.

Another aspect of diversity is geographical, as seen clearly in the summary of presentations by country shown in the figure below (Figure 1). This diversity is a recent phenomenon: I tried to organize an FBLD Symposium for Pacifichem 2005, but was unable to find enough participants from Pacific-rim countries, at least three of which must be represented among the organizers. Indeed, the first major FBLD-based event outside the US and Europe was held in Australia only in 2012 [5].

The articles in this issue illustrate both aspects of diversity, geographic as well as scientific. Ruan and colleagues from China provide an overview of the FBDD field [6]. Chemistry is covered in papers by Qingwen Zhang and colleagues in China [7], as well as Martin Scanlon and colleagues in Australia [8]. NMR is well-represented by papers from Ray Norton and colleagues in Australia [9] and Ivanhoe Leung and colleagues in New Zealand [10] and joins forces with modelling and chemistry in a paper by Thomas Leeper and colleagues in the US [11]. Another biophysical technique, mass spectrometry, is highlighted in a paper by Pedro and Quinn in Australia [12], while fragment-based approaches against bacterial pathogens are discussed by Begona Heras and colleagues, also from Australia [13].

The theme of the 2015 Pacifichem Meeting was Chemical Networking: Building Bridges Across the Pacific. I think everyone would agree that this goal was accomplished. It will be fun to revisit these bridges in 2020 to see how they have strengthened, and to see what new bridges have formed.

## Figures and Tables

**Figure 1:**
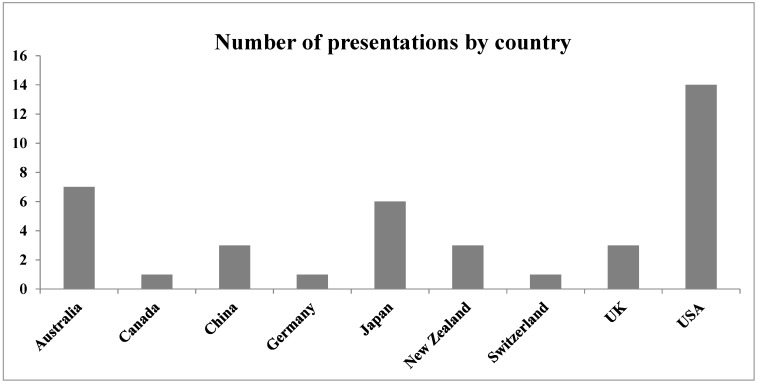
Number of presentations at the Pacifichem 2015 FBLD symposium arranged alphabetically by country.
